# Supplementary material for: Comet Assay Profiling of FLASH-Induced Damage: Mechanistic Insights into the Effects of FLASH Irradiation
Source: Int J Mol Sci. 2023 Apr 13;24(8):7195. doi: 10.3390/ijms24087195 (PMC10138874; doi:10.3390/ijms24087195)
Supplement: Supplementary file 1 [file ijms-24-07195-s001.zip › ijms-2279537-supplementary.pdf]

Supplementary Material:

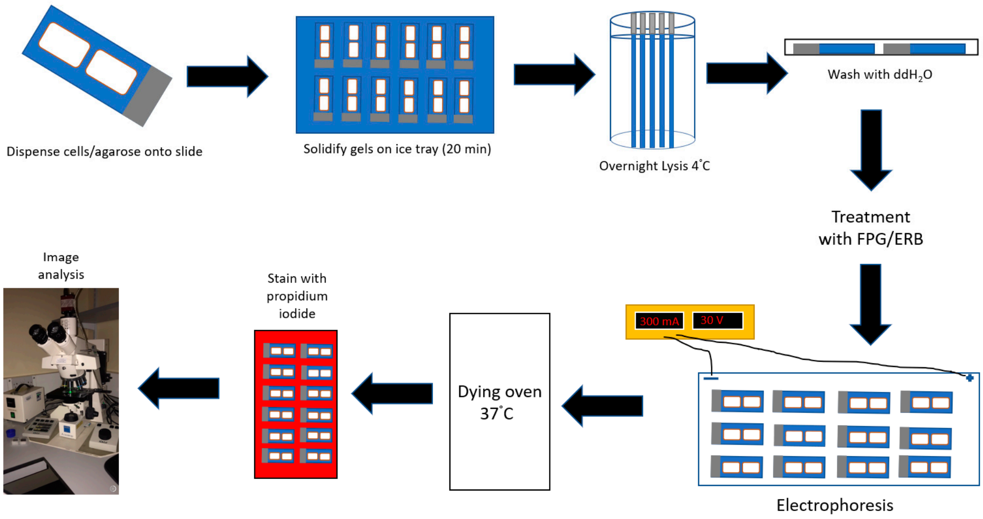

Figure S1. Alkaline Comet assay procedure flow diagram.

Cells (40,000) are obtained from an *in vitro* culture or *in vivo* test subject and mixed with 190 µl low melting point agarose at 37 °C with 80 µl of this mix being used to create a droplet of single cells on the surface of a glass microscope slide. A coverslip (22 x 22 mm) is placed on top allowing equal distribution of the molten agarose and the slide is placed onto a metal tray situated on flaked ice (4 °C) to allow formation of a gel.

These cells suspended in agarose may then be treated with DNA damaging agents i.e ionizing radiation and/or subject to overnight lysis. An alkaline solution with detergent and high salt are used to remove cell membrane/proteins producing tightly coiled nuclear DNA attached to a lysis resistant matrix. These 'nucleoid bodies' may be digested with lesion specific enzymes to induce further strand breaks at specific sites or washed with double distilled water before electrophoresis is performed at high pH. Supercoiled DNA loops containing a break lose their supercoiling and become free to extend towards the anode while unbroken DNA remains tightly coiled and its heavy molecular weight unable to traverse the gel. This results in structures resembling comets which may be observed by fluorescence microscopy and quantified by image analysis software.

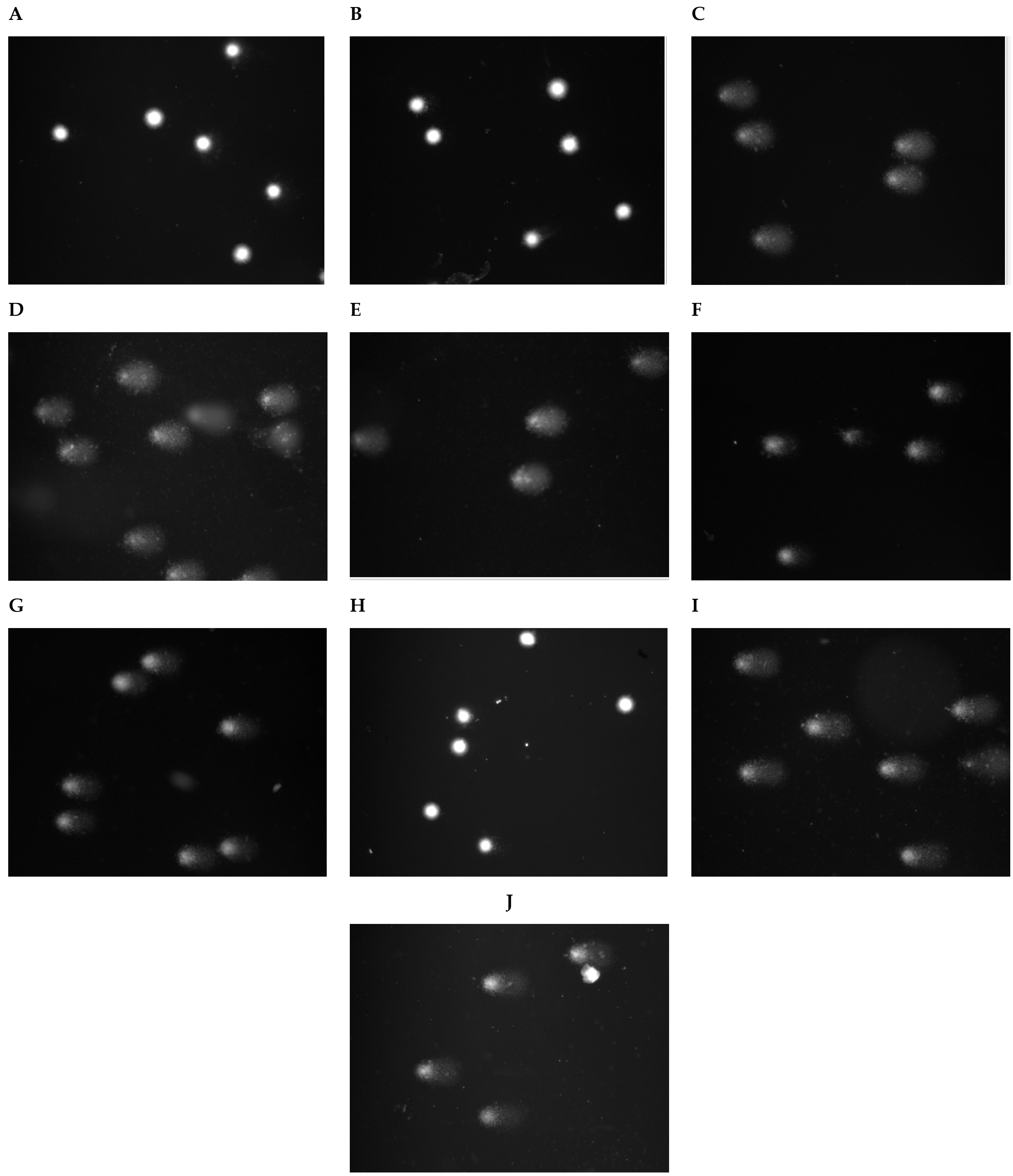

Figure S2. Representative images obtained from slides assessing FLASH-induced DNA interstrand crosslinks as a marker of radical + radical recombination. Images of whole blood peripheral blood lymphocytes (WB-PBL): **A)** unirradiated (0 Gy) equilibrated to 0.5% O_2. **B)** unirradiated (21% O_2 conditions). **C)** 20 Gy conventional (CONV, 0.1 Gy s^{-1}) irradiated (2 x 10 Gy) at 21% O_2. **D)** 20 Gy CONV irradiated at 0.5% O_2 and re-irradiated with CONV (10 Gy) at 21% O_2. **E)** FLASH (2 kGy s^{-1}) irradiated at 0.5% O_2 and re-irradiated with CONV (10 Gy) at 21% O_2. **F)** 20 Gy FLASH irradiated at 0.5% O_2. **G)** 20 Gy CONV irradiated at 0.5% O_2. **H)** treated with Cisplatin (1000 μM) unirradiated. **I)** irradiated to 10 Gy (0.026 Gy s^{-1}). **J)** treated with Cisplatin (1000 μM) and irradiated to 10 Gy (0.0026 Gy s^{-1}).

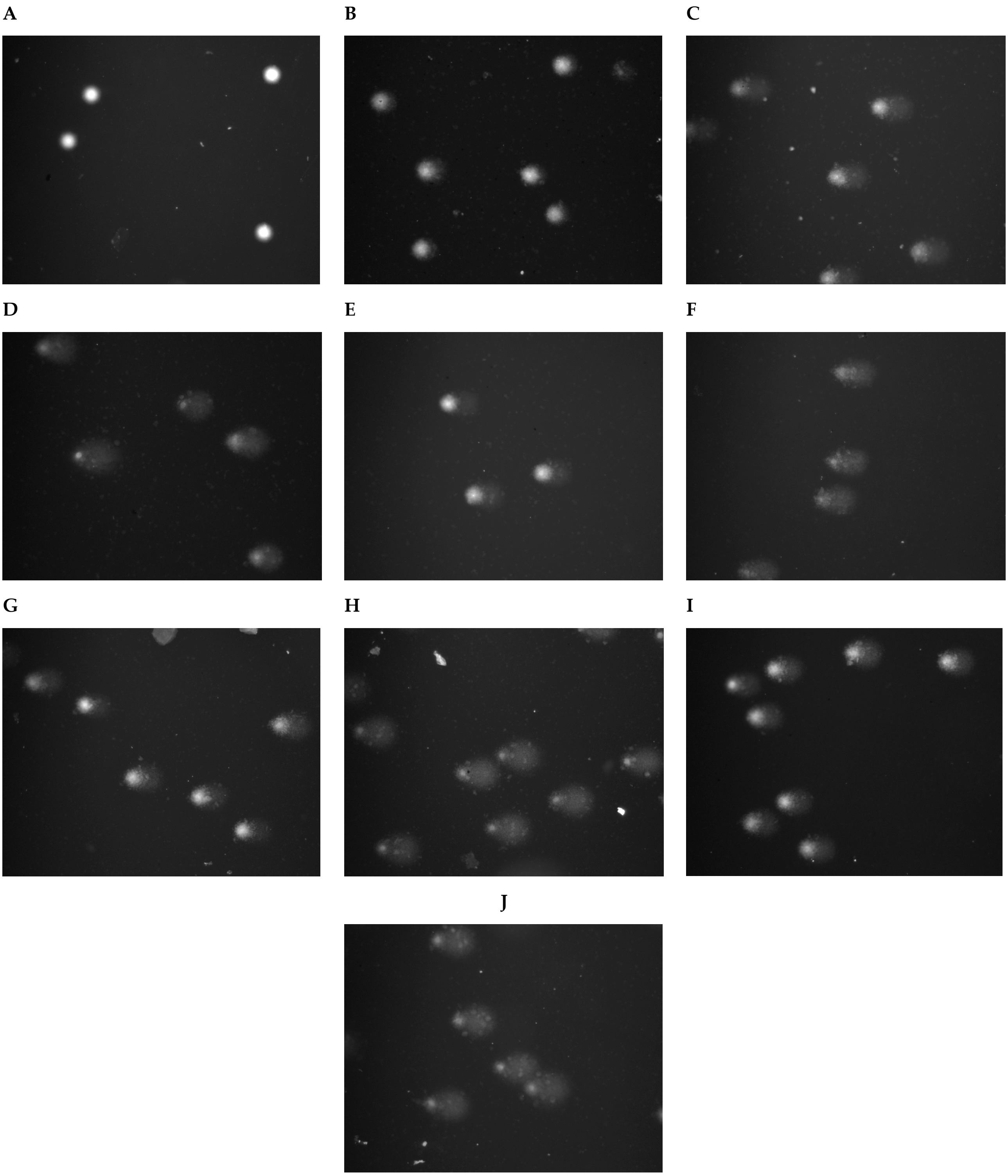

Figure S3. Representative images obtained from slides assessing FLASH-induced base vs. strand break damage as a possible marker of transient oxygen depletion. Images of whole blood peripheral blood lymphocytes (WB-PBL): **A)** unirradiated (0 Gy) equilibrated to 0.5% O_2 and treated with enzyme reaction buffer (ERB). **B)** unirradiated and equilibrated to 0.5% O_2 conditions treated with 0.1 U formamidopyrimidine-DNA glycosylase (FPG). **C)** 20 Gy conventional (CONV, 0.1 Gy s^{-1}) irradiated at 0.5% O_2 treated with ERB. **D)** 20 Gy CONV irradiated at 0.5% O_2 and treated with FPG. **E)** FLASH (2 kGy s^{-1}) irradiated at 0.5% O_2 treated with ERB. **F)** 20 Gy FLASH irradiated at 0.5% O_2 and treated with FPG. **G)** 20 Gy CONV irradiated at 0.35% O_2 treated with ERB. **H)** 20 Gy CONV irradiated at 0.35% O_2 treated with FPG. **I)** 20Gy CONV irradiated at 0.7% O_2 and treated with ERB. **J)** 20 Gy CONV irradiated at 0.7% and treated with FPG.

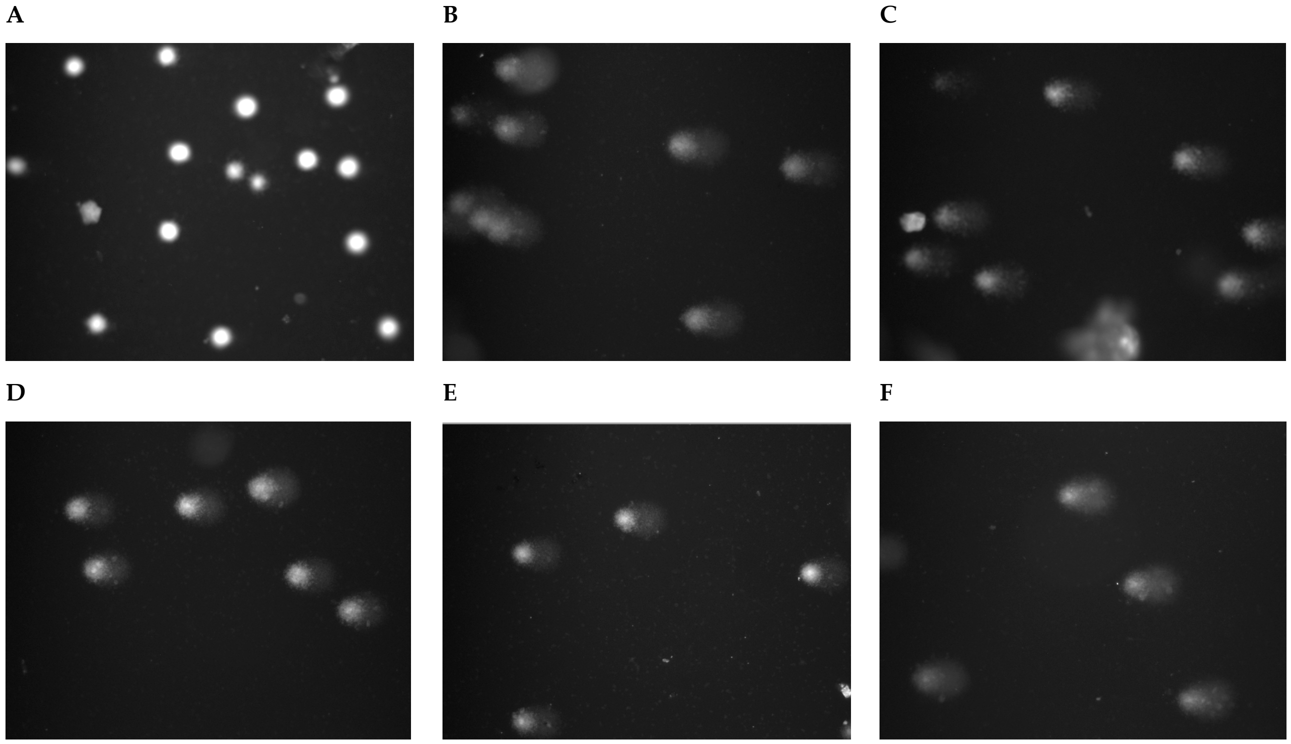

Figure S4. Representative images obtained from slides assessing BSO pre-treatment to evaluate the effect of lowering thiol levels on the FLASH-mediated reduced damage burden. Images of whole blood peripheral blood lymphocytes (WB-PBL): **A)** unirradiated (0 Gy) equilibrated to 0.5% O_2 treated with 5 mM buthionine sulfoximine (BSO) **B)** 20 Gy conventional (CONV, 0.1 Gy s^{-1}) irradiated at 0.5% O_2. **C)** 20 Gy CONV irradiated at 0.5% O_2 treated 5 mM BSO. **D)** 20 Gy FLASH (2 kGy s^{-1}) irradiated at 0.5% O_2. **E)** 20 Gy FLASH irradiated at 0.5% O_2 and treated with 5 mM BSO. **F)** 20 Gy CONV irradiated at 21% O_2.
